# Editorial: Discovery, development and implementation of improved options for treating opioid overdose in the synthetic opioid era

**DOI:** 10.3389/fpsyt.2024.1443304

**Published:** 2024-07-11

**Authors:** Christian Heidbreder, Mark K. Greenwald, Bernard Le Foll, Phil Skolnick

**Affiliations:** ^1^ Research & Development, Indivior, Inc., Chesterfield, VA, United States; ^2^ Department of Psychiatry and Behavioral Neurosciences, School of Medicine, Department of Pharmacy Practice, Eugene Applebaum College of Pharmacy and Health Sciences, Wayne State University, Detroit, MI, United States; ^3^ Translational Addiction Research Laboratory, Centre for Addiction and Mental Health, Toronto, ON, Canada

**Keywords:** opioid overdose crisis, synthetic opioids, fentanyl, carfentanil, naloxone, nalmefene, cardiac arrest and brain injuries, respiratory depression (RD)

Opioid overdose remains the leading cause of drug deaths in the United States (US), claiming 78,226 lives over the 12-month period ending December 2023. Approximately 92% (71,821) of opioid-related fatalities were linked to synthetic opioids (“synthetics”) (1). Synthetics are highly lipophilic molecules that rapidly partition into the central nervous system, which limits the window of opportunity for rescue following an overdose. This rapid onset of action, coupled with the high potencies and variable half-lives of synthetics, presents new challenges to reversing an overdose compared to opium-derived alkaloids (e.g., heroin, oxycodone). Furthermore, the ready availability of synthetics, often in the form of counterfeit pills (2) has resulted in an increase in fatal overdoses in opioid-naïve individuals including adolescents (3) and children younger than 6 years of age (4), who are now exposed to potentially lethal doses of synthetic opioids.

Although Naloxone HCl, which the US Food and Drug Administration (FDA) first approved in 1971, remains the mainstay for opioid overdose treatment, this Research Topic provides new insights into the urgency and importance of discovering, developing and implementing improved options for treating opioid overdose in the synthetic opioid era. Four manuscripts submitted to the journal were deemed suitable for publication after undergoing a thorough peer review process. The following summarizes the main results for each manuscript.

In the first article, *“Brain oxygen responses induced by opioids: focus on heroin, fentanyl, and their adulterants”*, Kiyatkin and Choi chronically implanted oxygen sensors in the rat nucleus accumbens and coupled them with high-speed amperometry to directly monitor brain oxygen responses induced by heroin and fentanyl. Both heroin and fentanyl were shown to produce a biphasic pattern of brain oxygen response: an initial transient decrease (hypoxia) followed by a subsequent weaker but more prolonged increase (hyperoxia), indicating the involvement of post-hypoxic compensatory vascular response. The hypoxic effects of heroin and fentanyl were potentiated or otherwise altered by the addition of alcohol, ketamine, and xylazine, further supporting a link between adulterated drug supply and heightened health complications. Finally, the authors provided new insights into the time-sensitive brain hypoxia induced by fentanyl and its relation to the timing of reversal by naloxone.

In the second article, *“Are carfentanil and acrylfentanyl naloxone resistant?”*, Feasel et al. applied *in vitro* techniques to establish the median effective inhibitory concentrations for fentanyl, acrylfentanyl, and carfentanil, and to evaluate naloxone’s efficacy in reversing agonist–receptor interactions. They first demonstrated that agonist actions of acrylfentanyl and carfentanil could be reversed with naloxone. Strikingly, although acrylfentanyl had approximately one-half the potency of fentanyl, this compound required nearly double the concentration of naloxone to reverse its agonist activity relative to fentanyl. Carfentanil, with potency ≈100× greater than fentanyl, required a significantly higher concentration of naloxone to antagonize a challenge of its EC_90_.

In the third article, *“Evaluating the rate of reversal of fentanyl-induced respiratory depression using a novel long-acting naloxone nanoparticle, cNLX-NP”*, Averick et al. characterized the efficacy of a novel opioid reversal agent based on covalent naloxone nanoparticles (cNLX-NP) to reverse fentanyl-induced respiratory effects, and the duration of its protective effects. The authors showed that cNLX-NP extended the terminal half-life of naloxone beyond that of naloxone alone or nalmefene, blocked fentanyl-induced respiratory depression up to 48 hours and rapidly reversed fentanyl-induced respiratory depression when combined 1:1 with free naloxone.

In the fourth article, *“Comparison of intranasal naloxone and intranasal nalmefene in a translational model assessing the impact of synthetic opioid overdose on respiratory depression and cardiac arrest”*, Laffont et al. used a validated translational model, which quantitatively predicts opioid-induced respiratory depression and cardiac arrest, to compare rates of fentanyl- and carfentanil-induced cardiac arrest events following rescue by intranasal (IN) administration of the mu-receptor antagonists naloxone and nalmefene. This model (5), developed by the FDA’s Division of Applied Regulatory Science, offers an unbiased approach to evaluating the effectiveness of these agents following a potentially lethal dose of synthetic opioid. Following simulated fentanyl- and carfentanil-induced overdoses in chronic opioid users, IN nalmefene substantially reduced the incidence of cardiac arrest compared to IN naloxone. Nalmefene also produced large and clinically meaningful reductions in the incidence of cardiac arrests in opioid-naïve subjects (see **Figure 1**). Across dosing scenarios, simultaneous administration of four doses of IN naloxone were needed to reduce the percentage of cardiac arrest events to levels produced by a single dose of IN nalmefene.

**Figure 1:**
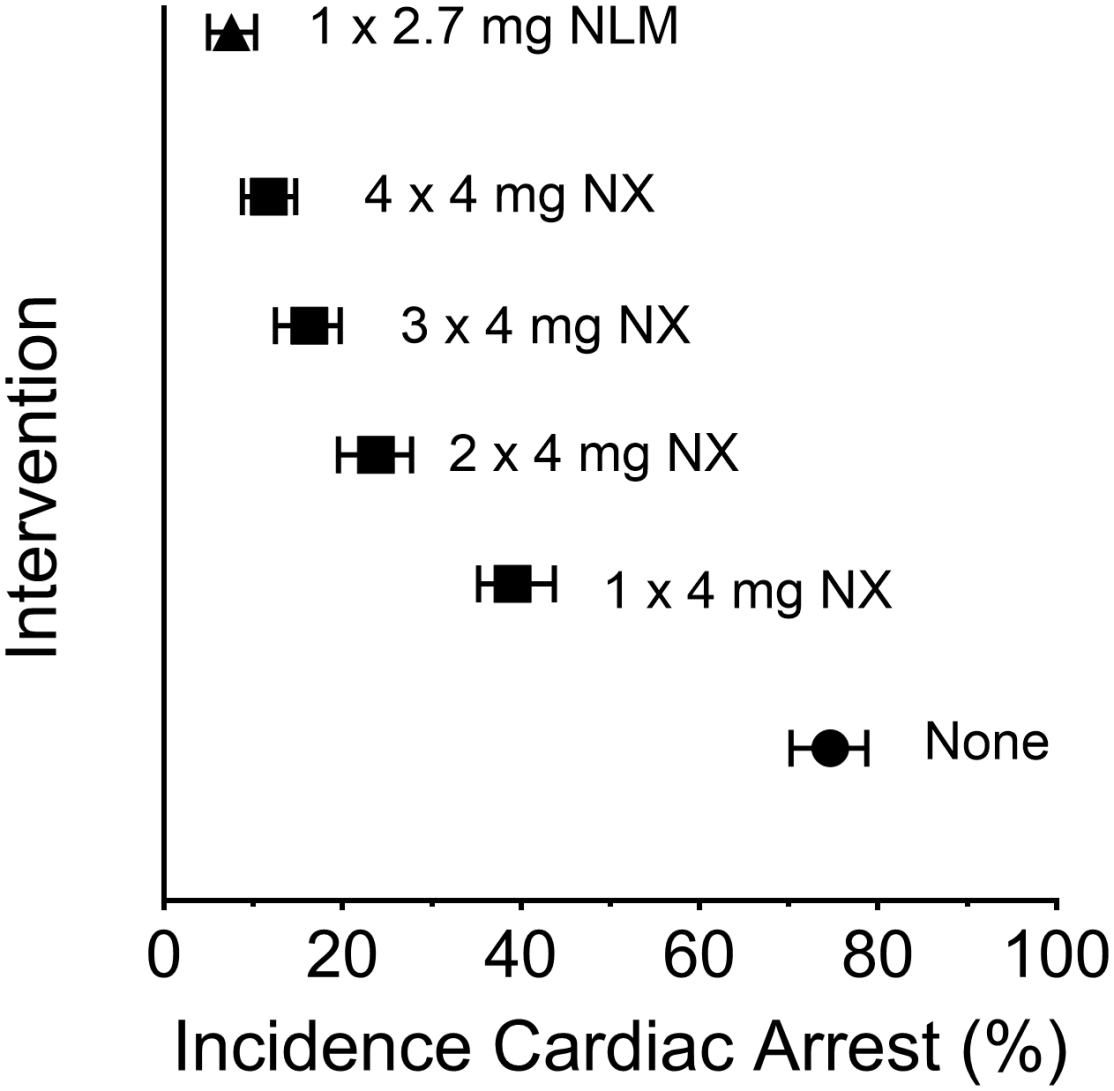
Incidence of cardiac arrest following a simulated intravenous overdose with fentanyl (1.63 mg): reversal by intranasal (IN) naloxone and IN nalmefene. Symbols represent the median percentage (and 95% confidence interval) of simulated subjects experiencing a cardiac arrest. The simulations were conducted using data from opioid-naïve individuals as described by Mann et al. (5); the data presented here are from Laffont et al. Legend: filled circle, none (no intervention following fentanyl); filled square, IN NX (4 mg intranasal naloxone); filled triangle, IN NLM (2.7 mg intranasal nalmefene, equivalent to 3 mg of nalmefene hydrochloride). Simultaneous administration of two, three or four doses of IN naloxone was simulated by administering a dose equal to 8 mg (2 x 4 mg), 12 mg (3 x 4 mg), or 16 mg (4 x 4 mg), respectively. The statistical approach for simulation was adapted from the model described by Mann et al. (5).

The editors thank all authors, reviewers, and editorial board members for contributing to this Research Topic. Recent developments in mathematical modeling, computational power, and availability of preclinical and clinical data sets are enabling the development of new mechanistic models to understand pharmacokinetic-pharmacodynamic interactions of new, fast-acting and potent opioid overdose reversal agents that may prevent enduring brain damage or death. We hope this Research Topic inspires innovative and life-saving research approaches in this field.

## Author contributions

CH: Conceptualization, Data curation, Formal analysis, Investigation, Methodology, Project administration, Supervision, Validation, Visualization, Writing – original draft, Writing – review & editing. MG: Conceptualization, Data curation, Formal analysis, Investigation, Methodology, Supervision, Validation, Visualization, Writing – review & editing. BF: Methodology, Validation, Writing – review & editing. PS: Conceptualization, Data curation, Formal analysis, Investigation, Methodology, Supervision, Validation, Visualization, Writing – review & editing.
